# Synthesis of Dinaphtho[2,3-*d*:2’,3’-*d*’]anthra[1,2-*b*:5,6-*b*’]dithiophene (DNADT) Derivatives: Effect of Alkyl Chains on Transistor Properties

**DOI:** 10.3390/ijms21072447

**Published:** 2020-04-01

**Authors:** Takumi Ishida, Yuta Sawanaka, Ryota Toyama, Zhenfei Ji, Hiroki Mori, Yasushi Nishihara

**Affiliations:** 1 Graduate School of Natural Science and Technology, Okayama University, 3-1-1 Tsushimanaka, Kita-ku, Okayama 700-8530, Japan; pimf2p6f@s.okayama-u.ac.jp (T.I.); pyhb7jum@s.okayama-u.ac.jp (Y.S.); pnah56kw@s.okayama-u.ac.jp (R.T.); puf77a9l@s.okayama-u.ac.jp (Z.J.); 2 Research Institute for Interdisciplinary Science, Okayama University, 3-1-1 Tsushimanaka, Kita-ku, Okayama 700-8530, Japan; h-mor@okayama-u.ac.jp

**Keywords:** organic field-effect transistor (OFET), thienoacene, p-type semiconductor, Negishi coupling reaction, cycloaromatization, fastener effect

## Abstract

To investigate organic field-effect transistor (OFET) properties, a new thienoacene-type molecule, 4,14-dihexyldinaphtho[2,3-*d*:2’,3’-*d*’]anthra[1,2-*b*:5,6-*b*’]dithiophene (C6-DNADT), consisting of π-conjugated nine aromatic rings and two hexyl chains along the longitudinal molecular axis has been successfully synthesized by sequential reactions, including Negishi coupling, epoxidation, and cycloaromatization. The fabricated OFET using thin films of C6-DNADT exhibited p-channel FET properties with field-effect mobilities (*µ*) of up to 2.6 × 10^−2^ cm^2^ V^−1^ s^−1^, which is ca. three times lower than that of the parent DNADT molecule (8.5 × 10^−2^ cm^2^ V^−1^ s^−1^). Although this result implies that the installation of relatively short alkyl chains into the DNADT core is not suitable for transistor application, the origins for the FET performance obtained in this work is fully discussed, based on theoretical calculations and solid-state structure of C6-DNADT by grazing incidence wide-angle X-ray scattering (GIWAXS) and atomic force microscopy (AFM) analyses. The results obtained in this study disclose the effect of alkyl chains introduced onto the molecule on transistor characteristics.

## 1. Introduction

Organic field-effect transistors (OFETs) using thin films and single crystals of π-extended thienoacene and thienophenacene molecules have attracted much attention as the key elements for realizing future ubiquitous electronics because they are known to display excellent hole transport properties [1,2,3,4,5]. In terms of single crystal OFETs, rubrene has provided excellent FET characteristics with carrier mobility (*μ*) as high as 40 cm^2^ V^–1^ s^–1^ [6], but the rubrene molecule has scarcely been available for thin-film FETs, i.e., very few rubrene thin-film FETs have been operated [7]. On the other hand, the highest *μ* of thin-film OFETs reported among the state-of-art materials is currently 43 cm^2^ V^−1^ s^−1^ for 2,7-dioctyl[1]benzothieno[3,2-*b*][1]benzothiophene (C8-BTBT) [8]. It has been reported that the *μ* value increases as the number of aromatic rings increases, i.e., more extension of the π-system is suitable for better transistor properties owing to their greater intermolecular overlaps in the molecular network [9]. The extension of the π-system derives relatively small reorganization energies (λ) that can lead to the high-performance OFET devices [10,11]. Furthermore, highest occupied molecular orbital (HOMO) of the molecules affects a stability of the fabricated devices upon oxidation [12]. For instance, the pentacene molecule is not air-stable owing to its relatively high HOMO energy level (–5.0 eV) [13]. 

The molecular modification by installation of alkyl chains onto the π-frameworks is expected to fasten the π-core of the molecule, based on forming a strong intermolecular stacking, which is called a ‘fastener effect’ [14]. Several examples have already been reported in alkylated picene [15], thienoacene [16,17], and thienophenacene [18,19]. In addition, the alkyl substitution of the molecules can enable the fabrication of solution-processed OFETs because their solubility in common organic solvents could be improved. In fact, the parent nine-ring-fused linear thienoacenes was not obtained due to its very low solubility, but dialkylated derivatives could be synthesized and their fabricated OFETs based on vapor-deposited thin film exhibited the hole mobility of up to 1 cm^2^ V^−1^ s^−1^ [20]. Hence, extremely π-extended polycyclic aromatic compounds bearing alkyl substituents are promising target molecules for high-performance OFET materials. However, the number of such compounds is still limited because of difficulty to synthesize the corresponding synthetic intermediates. 

**Figure 1:**
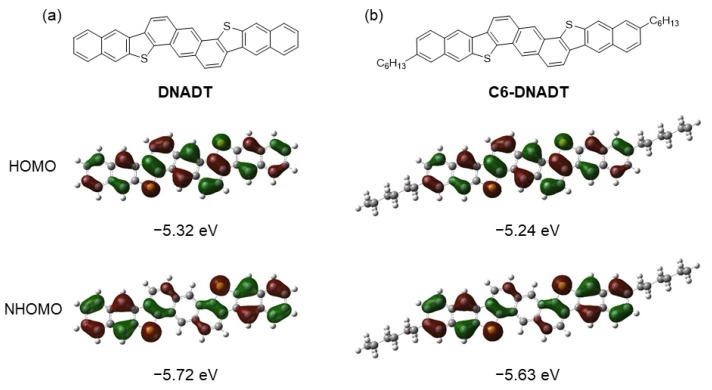
Molecular structures and highest occupied molecular orbital (HOMO) and next highest occupied molecular orbital (NHOMO) (B3LYP/6-311G(d)) of (**a**) dinaphtho[2,3-*d*:2’,3’-*d’*]anthra[1,2-*b*:5,6-*b’*]dithiophene (DNADT) (**1**) and (**b**) 4,14-dihexyldinaphtho[2,3-*d*:2’,3’-*d’*]anthra[1,2-*b*:5,6-*b’*]dithiophene (C6-DNADT) (**2**).

During our continuing studies on synthesis and characterization of an array of π-conjugated phenacene-type molecules for OFET properties [21,22,23,24,25,26,27,28,29,30,31], we have reported an efficient synthetic route and transistor properties of nine-ring-fused thienoacene molecule (dinaphtho[2,3-*d*:2’,3’-*d’*]anthra[1,2-*b*:5,6-*b’*]dithiophene (DNADT), Figure 1a) [32]. In addition, the fabricated OFET devices based on DNADT exhibited the hole mobility of up to 8.5 × 10^−2^ cm^2^ V^−1^ s^−1^. Expecting the improved OFET properties by the installation of alkyl chains onto the DNADT core, we herein report the new synthetic scheme and evaluation of the FET characteristics of dihexyl-substituted DNADT (4,14-dihexyldinaphtho[2,3-*d*:2’,3’-*d’*]anthra[1,2-*b*:5,6-*b’*]dithiophene (C6-DNADT), Figure 1b). Furthermore, the effect of alkyl-substitution on FET performance was investigated using grazing-incidence wide-angle X-ray scattering (GIWAXS)/atomic force microscopy (AFM) analyses as well as optical absorption spectroscopy. 

## 2. Results and Discussion

### 2.1. Theoretical Calculations for Molecular Design

The positions to introduce alkyl chains in the molecular framework is an important issue for improvement of OFET properties of the materials. In some cases, the installation of alkyl substituents along the longitudinal molecular axis dramatically increased carrier mobility [33,34]. Thereby, we designed the molecule having two hexyl groups in 4,14-positions, expecting a good balance between crystallinity and solubility. Based on density functional theory (DFT) calculations at the B3LYP/6-311G(d) level [35], as shown in Figure 1, it is found that 4,14-dihexyldinaphtho[2,3-*d*:2′,3′-*d*’]anthra[1,2-*b*:5,6-*b’*]dithiophene (C6-DNADT) had the HOMO and second (next) highest occupied molecular orbital (NHOMO), with very similar characteristics to those in DNADT that has large coefficients on two sulfur atoms, leading to effective orbital overlaps through sulfur-sulfur (S–S) interactions [36,37,38]. Based on the band transport model for organic semiconductors, the strength of hole-vibration coupling (or hole-phonon coupling (h-ph coupling)) between HOMOs is essential [39]. In contrast, energy levels of two molecules are slightly different; the estimated HOMO energy level of C6-DNADT was –5.24 eV, which is slightly higher than that of DNADT (–5.32 eV), expecting that C6-DNADT might realize a lower voltage operation (|*V*_th_|) than DNADT. Recently, Kobayashi reported that NHOMO effects on the valence band structure of organic semiconductors [40]. The energy gap between HOMO and NHOMO of C6-DNADT (0.39 eV) is comparable to that of DNADT (0.40 eV). 

### 2.2. Synthesis of C6-DNADT

**Scheme 1:**
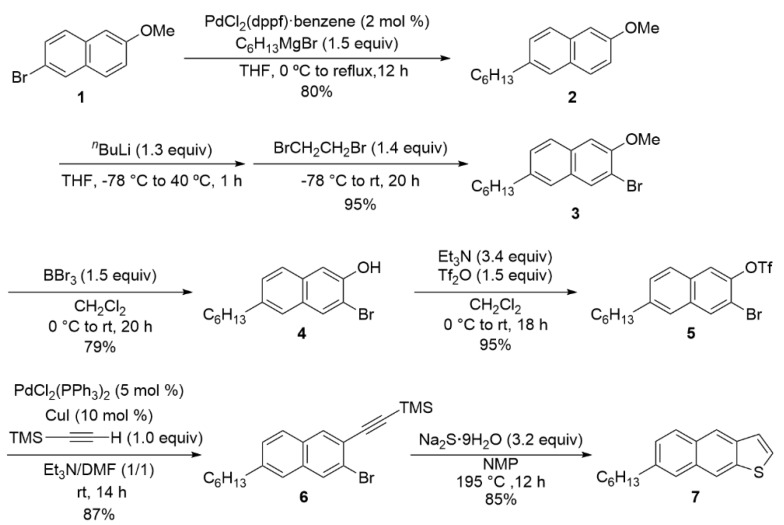
Synthetic route of naphtho[2,3-*b*]thiophene (**7**).

First, we considered how to introduce alkyl chains into the DNADT framework. Starting from commercially available 2-bromo-6-methoxynaphthalene (**1**), one of coupling partners, 6-hexylnaphtho[2,3-*b*]thiophene (**7**) was synthesized according to the synthetic method for anthra[2,3-*b*]thiophene (Scheme 1) [41]. The palladium-catalyzed Kumada–Tamao–Corriu coupling of **1** with hexyl Grignard reagent afforded **2**. Successively, regioselective bromination at the 3-position of naphthalene via lithiation gave 3-bromo-6-hexyl-2-methoxynaphthalene (**3**), which was demethylated with boron tribromide to afford 3-bromo-6-hexylnaphthalen-2-ol (**4**). Then, a hydroxy group of **4** was converted into the corresponding triflate **5**, which was then utilized in Sonogashira–Hagihara coupling with trimethylsilylethyne to afford the precursor **6**. The excellent chemoselective alkynylation for the Sonogashira–Hagihara coupling at a triflate over a bromine moiety of **6** was achieved using *N*,*N*-dimethylformamide (DMF) as the solvent. Finally, thienoannulation reaction was accomplished with sodium sulfide nonahydrate (Na_2_S·9H_2_O) to give the target product **7** in 85% isolated yield. It is noteworthy that since the common starting compound **1** can be commercially available, the synthesis of other types of alkylated derivatives of compound **7** could be possible. 

**Scheme 2:**
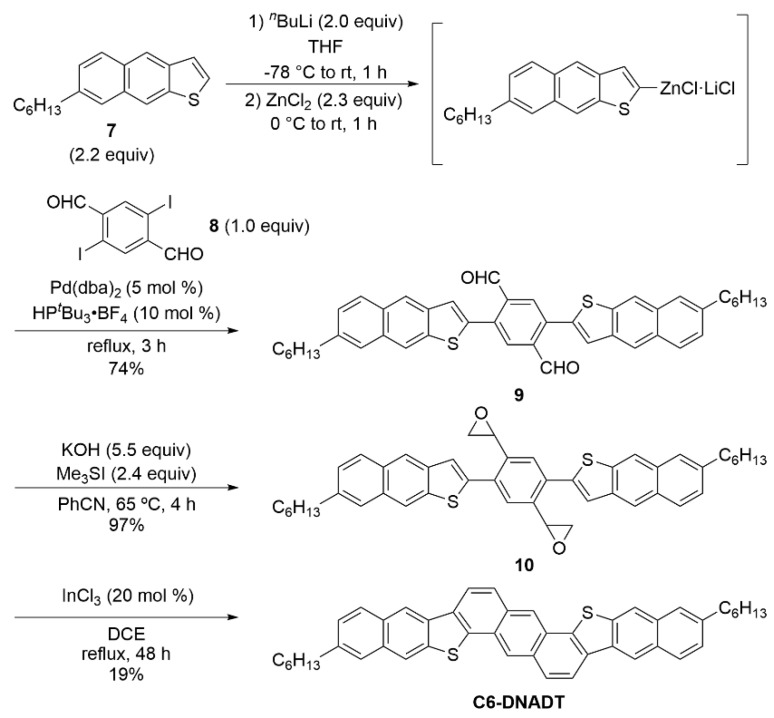
Synthetic route of C6-DNADT.

The synthetic route of C6-DNADT from compound **7** is illustrated in Scheme 2. This 3-step synthetic method has been established by us [18,19]. First, palladium-catalyzed Negishi coupling of organozinc reagent, prepared *in situ* by lithiation of **7** by treatment with *n*-BuLi followed by zincation, with **8** afforded dialdehyde **9** in 74% yield. Subsequently, epoxidation of **9** and a sequential indium-catalyzed intramolecular cycloaromatization of **10** gave C6-DNADT as an orange solid, albeit in 19% yield. Unexpectedly, even though two hexyl chains were introduced onto the DNADT core, solubility of C6-DNADT was found to be very poor, which is unable to measure NMR in solution. To prepare a pure sample suitable for further evaluation of the physicochemical and FET properties, the synthesized C6-DNADT was further purified twice by a gradient vacuum sublimation. 

### 2.3. Physicochemical Properties of C6-DNADT

#### 2.3.1. UV-Vis Absorption Spectrum and Cyclic Voltammogram

**Figure 2:**
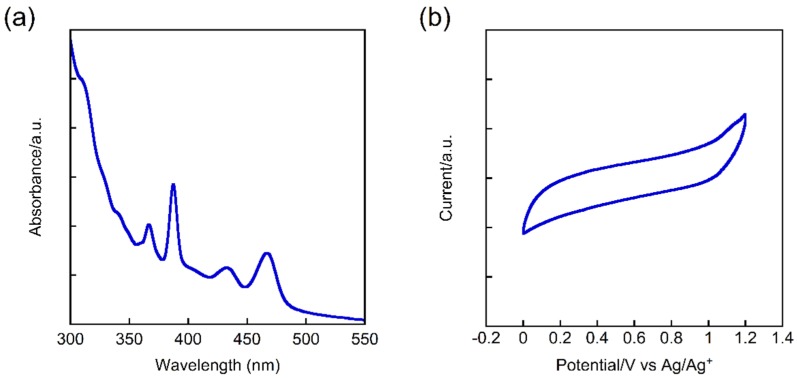
(**a**) UV-Vis absorption spectrum in thin film and (**b**) cyclic voltammogram in CH_2_Cl_2_ solution of C6-DNADT.

To evaluate physicochemical properties of C6-DNADT, UV-vis absorption spectrum was measured for its vapor-deposited thin film (Figure 2a). The maximum absorption was observed at 467 nm and the optical energy gap estimated from an absorption edge was 2.48 eV, which is similar to that of DNADT (2.51 eV) [32], indicating that the introduction of alkyl groups did not affect its optical energy gap in thin film. However, the two obvious peaks appeared at 367 and 388 nm in thin film of C6-DNADT and the shape of UV-vis absorption spectrum in C6-DNADT is quite different from that of the parent DNADT, implying the formation of different structure in the solid state. 

Cyclic voltammogram of C6-DNADT in dichloromethane solution was measured to estimate its frontier energy level (Figure 2b). C6-DNADT showed a very weak oxidation wave with oxidation onset (*E*^ox^_onset_) of +1.02 V (vs. Ag/Ag^+^) due to its poor solubility. The estimated HOMO energy level of C6-DNADT was −5.29 eV, which is similar to that of the result of DFT calculation (Figure 1). As expected, this HOMO energy level is close to the work function of gold (5.1 eV) [42], which could be expected to lead to the smooth hole injection in OFETs [43]. In addition, this HOMO value is sufficiently deep to achieve the high air-stability. Thus, C6-DNADT-based OFET may show the good transistor property under ambient conditions.

#### 2.3.2. Thermal Stability

**Figure 3:**
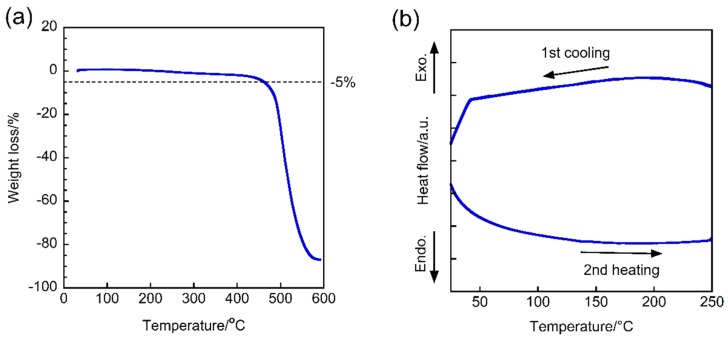
(**a**) Thermogravimetric analysis (TGA) and (**b**) differential scanning calorimetry (DSC) charts of C6-DNADT.

In order to evaluate a thermal stability of C6-DNADT, thermogravimetric analysis (TGA) and differential scanning calorimetry (DSC) were measured (Figure 3). The temperature of 5% weight loss (*T*_d_^5^) was 462 °C. Furthermore, no transition peaks were observed of up to 270 °C in the DSC curve, despite having the two flexible alkyl chains. These results indicate that C6-DNADT has high thermal stability due to a high rigidity and a large π-extended electron system of the DNADT core, which is beneficial for practical application in OFETs. 

#### 2.3.3. OFET Properties

**Figure 4:**
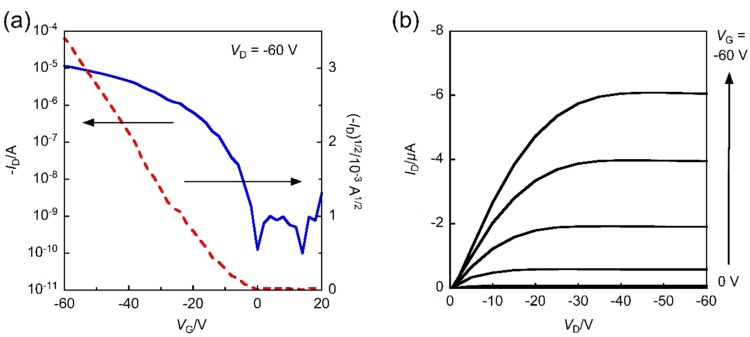
(**a**) Transfer and (**b**) output curves of C6-DNADT-based investigate organic field-effect transistor (OFET).

To investigate FET properties of C6-DNADT, typical bottom-gate top-contact devices based on its thin films have been fabricated using SiO_2_ gate dielectrics with the channel length (*L*) of 87 μm and width (*W*) of ca. 1980 μm. The surface of the n^+^-Si/SiO_2_ substrate was treated with *n*-octyltrichlorosilane (OTS) or *n*-octadecyltrichlorosilane (ODTS) as the self-assembled monolayer (SAM). The active layers were deposited on the SAM treated substrate by vapor deposition at a rate of 0.5 Å s^−1^ under reduced pressure (5 × 10^−5^ Pa). The substrate temperature was room temperature. Thermal annealing of the active layer was performed at 50, 100, and 150 °C for 30 min under an inert atmosphere. The measurements were conducted under ambient conditions in the dark. The transfer and output curves are shown in Figure 4 and the obtained FET properties, including anneal temperatures, hole mobility (*μ)*, threshold voltage (*V*_th_), and on-off ratio (*I*_on_/*I*_off_), are summarized in Table 1. Typical p-channel FET properties were observed in the transfer and output curves of all devices. As we expected, C6-DNADT-based OFETs exhibited smaller threshold voltage (*V*_th_) than that of DNADT-based devices due to its high-lying HOMO energy level [32]. In the case of ODTS-treated OFETs, the OFET based on as-deposited thin film showed hole mobility of up to 1.4 x 10^−2^ cm^2^ V^−1^ s^−1^. Further increasing the temperature of a thermal annealing process did not enhance the hole mobility. With OTS as the SAM, all devices exhibited higher hole mobilities than that of the corresponding ODTS-treated OFETs. The FET device without a thermal annealing exhibited hole mobility of 1.9 × 10^−2^ cm^2^ V^−1^ s^−1^, whereas a thermal annealing at 100 °C improved the maximum mobility of up to 2.6 × 10^−2^ cm^2^ V^−1^ s^−1^, which is highest hole mobility in this system. However, the obtained hole mobility of C6-DNADT was still lower than that of DNADT (*μ* = 8.5 × 10^−2^ cm^2^ V^−1^ s^−1^) [32]. The reason why the installation of alkyl chains providing such worse FET characteristics is discussed based on the topological and electronic features of its thin film. 

#### 2.3.4. AFM Images

**Figure 5:**
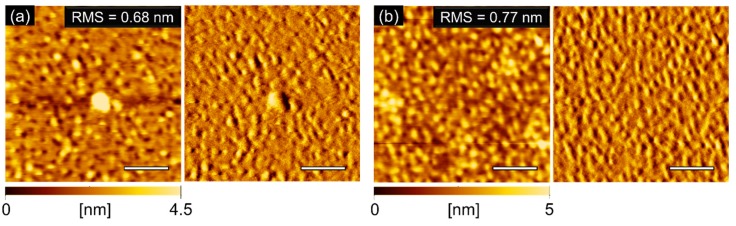
Topographic (left) and error-signal (right) images (2 *μ*m × 2 *μ*m) of thin films of C6-DNADT on octyltrichlorosilane (OTS)-modified n^+^-Si/SiO_2_ substrate; (**a**) *T*_anneal_ = as-deposited and (**b**) *T*_anneal_ = 100 °C. The scale bar is 500 nm.

To investigate the thin-film structure of C6-DNADT, we performed an atomic force microscope (AFM) analysis of the vapor-deposited thin film. AFM images of thin films as-deposited and annealed at 100 °C are shown in Figure 5. Obviously, the two surface morphologies were quite different. In as-deposited thin film, no distinct domain, many dark spots, and the smooth surface with root-mean-square (RMS) of 0.68 nm was observed. In contrast, thin film treated by a thermal annealing at 100 °C formed well-defined domain and has slightly higher roughness of RMS = 0.77 nm, resulting in the highest hole mobility due to its appropriate morphology. However, even thin film of C6-DNADT treated by thermal annealing at 100 °C has drastically smaller domain size (ca. 80 nm) than that of parent DNADT (ca. 300–500 nm) [32]. Moreover, many grain boundaries were also found, leading to its poor interlayer connectivity, which may inhibit an effective carrier transport [44]. Thus, C6-DNADT-based OFET exhibited poor hole mobility than that of the DNADT-based devices. 

#### 2.3.5. GIWAXS Images

**Figure 6:**
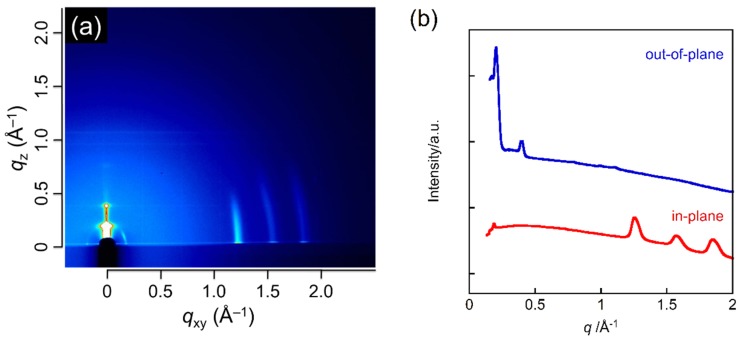
(**a**) Two-dimensional (2D) GIWAXS image and (**b**) one-dimensional (1D) profiles of GIWAXS patterns of C6-DNADT thin film on OTS-modified n^+^-Si/SiO_2_ substrate (*T*_anneal_ = 100 °C).

To further understand the difference of OFET performances between the parent DNADT and C6-DNADT, we investigated the grazing incidence wide-angle X-ray scattering (GIWAXS) analysis in thin film. Two-dimensional (2D) GIWAXS image and one-dimensional (1D) profiles extracted from GIWAXS image are shown in Figure 6. In the *q*_z_ direction, two series of (00*l*) diffractions were observed. The calculated interlayer distance (*d*_001_) was 30.7 Å, which is smaller than a molecular length estimated from a theoretical calculation (35.9 Å, Figure 1). Therefore, C6-DNADT is tilted at an angle of 31° with respect to the substrate. Furthermore, in the *q*_xy_ axis direction, three characteristic reflections were observed at 1.25, 1.57, and 1.84 Å^−1^, implying that C6-DNADT forms a herringbone structure as similar to that of the parent DNADT [32]. Although a weak (001) diffraction was also observed at the *q*_xy_ direction, indicating a contamination of an unsuitable face-on crystallite, the intensity of this face-on crystallite was much weaker than that of the parent DNADT. This result suggests that the introduction of two alkyl side chains along the longitudinal molecular axis can suppress the construction of the unfavorable face-on crystallite. However, the diffraction intensity of C6-DNADT was obviously weaker than that of DNADT. Such low crystalline nature is consistent with the result of AFM images, leading to the lower hole transporting ability than that of DNADT. One possible reason for such low crystalline nature of C6-DNADT might be attributed to the length of alkyl side chains. In general, the introduction of alkyl side chains along the longitudinal molecular axis can enhance the construction of densely packing structure owing to hydrophobic interaction (i.e., a fastener effect) [14]. However, the length of two hexyl side chains in C6-DNADT is much shorter than that of the central DNADT core. In this case, a fastener effect is not sufficient, because a hydrophobic interaction would be small [45]. Thus, the introduction of alkyl side chains with an appropriate length is highly important to develop the high-performance organic semiconductors for FET applications. 

## 3. Materials and Methods 

### 3.1. Instrumentation

All the reactions were carried out under an Ar atmosphere using standard Schlenk techniques. Glassware was dried in an oven (130 °C) and heated under reduced pressure prior to use. For thin layer chromatography (TLC) analyses throughout this work, Merck pre-coated TLC plates (silica gel 60 GF_254_, 0.25 mm) were used. Silica gel column chromatography was carried out using silica gel 60 N (spherical, neutral, 40−100 μm) from Kanto Chemicals Co., Ltd. The ^1^H and ^13^C{^1^H} NMR spectra were recorded on a Varian Mercury-300 (300 MHz), Varian 400-MR (400 MHz), and Varian INOVA-600 (600 MHz) spectrometer (Supplementary Materials). High-resolution mass spectrometry (HRMS) was carried out on a JEOL JMS-700 MStation (double-focusing mass spectrometer). Elemental analyses were carried out with a PerkinElmer 2400 CHN elemental analyzer at Okayama University. Infrared spectra were recorded on a Shimadzu IRPrestige-21 spectrophotometer and reported in wave numbers (cm^−1^). UV-vis absorption spectra were measured using a Shimadzu UV-2450 UV-vis spectrometer. Differential scanning calorimetry (DSC) measurement was performed at the rate of 10 °C/min from 25 °C to 270 °C for both heating and cooling steps under a nitrogen flow using a SSC5200H (Seiko Instruments). Thermogravimetric analysis (TGA) was carried out at a heating rate of 10 °C/min from 25 °C to 600 °C under a nitrogen flow rate of 20 mL/min using a TG4000 (Perkin Elmer). Dynamic force-mode atomic force microscopy (AFM) was carried out using an SPA 400-DFM (SII Nano Technologies). Grazing incidence wide-angle X-ray scattering (GIWAXS) analysis was performed at the SPring-8 on beamline BL46XU. The sample was irradiated at a fixed angle on the order of 0.12° through a Huber diffractometer with an X-ray energy of 12.39 keV (λ = 1 Å), and the GIWAXS patterns were recorded with a 2D image detector (Pilatus 300K). The thin films of C6-DNADT were fabricated by vapor deposition on OTS or ODTS-treated n^+^-Si/SiO_2_ substrate. The FET properties were measured at room temperature in air on a Keithley 6430 subfemtoampere remote source meter combined with a Keithley 2400 measure-source unit. Geometry optimizations and normal-mode calculations were performed at the B3LYP/6-311G(d) level using the Gaussian 09, Revision D. 01, program package. 

### 3.2. Chemicals

All other chemicals were used without further purification unless otherwise noted. 2-Bromo-6-methoxynaphthalene (**1**) (TCI), *n*-butyllithium (TCI), 1,2-dibromoethane (TCI), boron tribromide (TCI), trifluoromethanesulfonic anhydride (TCI), ethynyltrimethylsilane (Aldrich), copper(I) iodide (Nacalai Tesque), sodium sulfide nonahydrate (Nacalai Tesque), 1,4-benzenedimethanol (TCI), acetic anhydride (Wako), iodine (Nacalai Tesque), orthoperiodic acid (Wako), sodium hydroxide (Nacalai Tesque), pyridinium chlorochromate (TCI), zinc chloride (TCI), trimethylsulfonium iodide (Aldrich), potassium hydroxide (Nacalai Tesque), and indium trichloride (TCI) were purchased and used as received. Moreover, 2,5-Diiodo-1,4-benzendicarboxaldehyde (**2**) [46] was prepared according to the synthetic procedure and exhibited the identical spectra reported in the literature. 

### 3.3. Experimental Procedures

#### 3.3.1. Synthesis of 2-Hexyl-6-methoxynaphthalene (**2**) 

To a solution of 2-bromo-6-methoxynaphthalene (**1**) (1.42 g, 5.99 mmol, 1 equivalent (equiv)) and PdCl_2_(dppf)·benzene (97.2 mg, 0.12 mmol, 2 mol %) in dehydrated THF (35 mL) in 50 mL of Schlenk tube, was added dropwise hexylmagnesium bromide (1.13 M in THF, 8.0 mL, 9.0 mmol, 1.5 equiv) at 0 °C. The resulting mixture was refluxed for 12 h. After the reaction mixture was returned to room temperature, the reaction was quenched with saturated (sat.) NH_4_Cl aqueous (aq.) solution and extracted with EtOAc (50 × 3 mL). The combined organic layers were washed with brine and dried over MgSO_4_. After evaporating the volatiles under reduced pressure (170 Torr, 40 °C), the residue was purified by silica gel column chromatography (hexane:EtOAc = 5:1) to give **2** (1.42 g, 4.76 mmol) in 80% yield as a white solid. *R*_f_ = 0.83. ^1^H NMR (600 MHz, CDCl_3_, rt): *δ* 0.88 (t, *J* = 6.9 Hz, 3H), 1.30–1.37 (m, 6H), 1.67–1.69 (m, 2H), 2.73 (t, *J* = 7.5 Hz, 2H), 3.91 (s, 3H), 7.11–7.12 (m, 2H), 7.30 (dd, *J* = 8.1 Hz, *J* = 1.5 Hz, 1H), 7.54 (s, 1H), 7.66 (dd, *J* = 9.0 Hz, *J* = 6.6 Hz, 2H). 

#### 3.3.2. Synthesis of 3-Bromo-6-hexyl-2-methoxynaphthalene (**3**)

To a solution of **2** (1.68 g, 5.63 mmol) in dehydrated THF (12.5 mL) in 50 mL of Schlenk tube, was added dropwise *^n^*BuLi (1.6 M in hexane, 4.5 mL, 7.2 mmol, 1.3 equiv) at –78 °C. The resulting mixture was stirred at 40 °C. After 1 h, 1,2-dibromoethane (0.7 mL, 7.88 mmol, 1.4 equiv) was added at –78 °C, and the mixture was stirred at room temperature for 20 h. The reaction mixture was quenched with water, and extracted with CHCl_3_ (50 × 3 mL). The combined organic layers were washed with brine, and dried over MgSO_4_. After the volatiles were removed under vacuum (170 Torr, 40 °C), the residue was purified by silica gel column chromatography (hexane:EtOAc = 20:1) to give **3** (1.72 g, 5.35 mmol) in 95% yield as a white solid. *R*_f_ = 0.71. Mp: 60–62°C. FT-IR (KBr, cm^−1^): 2926 (s), 2852 (s), 1221 (s), 1041 (s). ^1^H NMR (400 MHz, CDCl_3_, rt): *δ* 0.89(t, *J* = 6.8 Hz, 3H), 1.33–1.38 (m, 6H), 1.66–1.70 (m, 2H), 2.73 (t, *J* = 7.8 Hz, 2H), 3.99 (s, 3H), 7.13 (s, 1H), 7.31 (dd, *J* = 8.6 Hz, *J* = 1.4 Hz, 1H), 7.45 (s, 1H), 7.64 (d, *J* = 8.4 Hz, 1H), 7.99 (s, 1H); ^13^C{^1^H} NMR (150 MHz, CDCl_3_, rt): *δ* 14.2, 22.8, 29.1, 31.5, 31.9, 36.0, 56.3, 106.7, 113.3, 125.3, 126.6, 128.4, 129.8, 131.9, 132.0, 139.3, 153.1. Anal. Calcd for C_16_H_19_BrO: C, 63.56; H, 6.59%. Found: C, 63.59; H, 6.61%. 

#### 3.3.3. Synthesis of 3-Bromo-6-hexylnaphthalen-2-ol (**4**)

To a solution of **3** (1.66 g, 5.17 mmol) in dehydrated CH_2_Cl_2_ (20.8 mL) in 50 mL of Schlenk tube, was added dropwise boron tribromide (1.0 M in CH_2_Cl_2_, 7.8 mL, 7.8 mmol, 1.5 equiv) at 0 °C. The resulting mixture was stirred at room temperature for 20 h. The reaction was quenched with water, and extracted with CH_2_Cl_2_ (50 × 3 mL). The combined organic layers were washed with brine, and dried over MgSO_4_. After the volatiles were evaporated in vacuo (350 Torr, 40 °C), the residue was purified by silica gel column chromatography (hexane:EtOAc = 10:1) to give **4** (1.26 g, 4.10 mmol) in 79% yield as a white solid. *R*_f_ = 0.38. Mp 59–60°C. FT-IR (KBr, cm^−1^): 3523 (br), 3207 (br), 2924 (s), 2850 (m), 1230 (m). ^1^NMR (400 MHz, CDCl_3_, rt): *δ* 0.88 (t, *J* = 6.8 Hz, 3H), 1.31–1.37 (m, 6H), 1.61–1.69 (m, 2H), 2.72 (t, *J* = 7.7 Hz, 2H), 5.53 (s, 1H), 7.30 (dd, *J* = 4.6 Hz, *J* = 1.7Hz, 1H), 7.35 (s, 1H), 7.45 (s, 1H), 7.61 (d, *J* = 8.4 Hz, 1H), 7.95 (s, 1H); ^13^C{^1^H} NMR (150 MHz, CDCl_3_, rt): *δ* 14.2, 22.8, 29.1, 31.4, 31.9, 36.0, 110.7, 112.5, 125.2, 126.6, 128.7, 129.8, 130.8, 132.5, 139.2, 148.9. HRMS (FAB^+^, *m*/*z*): [M]^+^ calcd for C_16_H_19_BrO_2_, 306.0619; found, 306.0612. 

To a solution of **3** (1.66 g, 5.17 mmol) in dehydrated CH_2_Cl_2_ (20.8 mL) in 50 mL of Schlenk tube, was added dropwise boron tribromide (1.0 M in CH_2_Cl_2_, 7.8 mL, 7.8 mmol, 1.5 equiv) at 0 °C. The resulting mixture was stirred at room temperature for 20 h. The reaction was quenched with water, and extracted with CH_2_Cl_2_ (50 × 3 mL). The combined organic layers were washed with brine, and dried over MgSO_4_. After the volatiles were evaporated in vacuo (350 Torr, 40 °C), the residue was purified by silica gel column chromatography (hexane:EtOAc = 10:1) to give **4** (1.26 g, 4.10 mmol) in 79% yield as a white solid. *R*_f_ = 0.38. Mp 59–60°C. FT-IR (KBr, cm^−1^): 3523 (br), 3207 (br), 2924 (s), 2850 (m), 1230 (m). ^1^NMR (400 MHz, CDCl_3_, rt): *δ* 0.88 (t, *J* = 6.8 Hz, 3H), 1.31–1.37 (m, 6H), 1.61–1.69 (m, 2H), 2.72 (t, *J* = 7.7 Hz, 2H), 5.53 (s, 1H), 7.30 (dd, *J* = 4.6 Hz, *J* = 1.7Hz, 1H), 7.35 (s, 1H), 7.45 (s, 1H), 7.61 (d, *J* = 8.4 Hz, 1H), 7.95 (s, 1H); ^13^C{^1^H} NMR (150 MHz, CDCl_3_, rt): *δ* 14.2, 22.8, 29.1, 31.4, 31.9, 36.0, 110.7, 112.5, 125.2, 126.6, 128.7, 129.8, 130.8, 132.5, 139.2, 148.9. HRMS (FAB^+^, *m*/*z*): [M]^+^ calcd for C_16_H_19_BrO_2_, 306.0619; found, 306.0612. 

#### 3.3.4. Synthesis of 3-Bromo-6-hexyl-2-(trifluoromethanesulfonyloxy)naphthalene (**5**)

To a solution of **4** (1.63 g, 5.31 mmol) and triethylamine (2.5 mL, 17.9 mmol, 3.4 equiv) in dehydrated CH_2_Cl_2_ (38 mL) in 50 mL of Schlenk tube, was added dropwise trifluoromethanesulfonic anhydride (1.3 mL, 7.7 mmol, 1.5 equiv) at 0 °C. The resulting mixture was stirred at room temperature for 18 h. The reaction mixture was quenched with water, and extracted with CH_2_Cl_2_ (50 × 3 mL). The combined organic layers were washed with brine, and dried over MgSO_4_. After the volatiles were removed (400 Torr, 40 °C), the residue was purified by silica gel column chromatography (hexane:EtOAc = 10:1) to give **5** (2.21 g, 5.04 mmol) in 95% yield as a yellow oil. *R*_f_ = 0.88. FT-IR (KBr, cm^−1^): 2930 (s), 2856 (s), 1427 (s), 1213 (s), 1139 (s). ^1^H NMR (600 MHz, CDCl_3_, rt): *δ* 0.88 (t, *J* = 7.0 Hz, 3H), 1.30–1.36 (m, 6H), 1.66–1.70 (m, 2H), 2.77 (t, *J* = 7.8 Hz, 2H), 7.44 (dd, *J* = 8.6 Hz, *J* = 1.8 Hz, 1H), 7.56 (s, 1H), 7.76 (d, *J* = 8.4 Hz, 2H), 8.10 (s, 1H); ^13^C{^1^H} NMR (150 MHz, CDCl_3_, rt): *δ* 14.2, 22.7, 29.1, 31.2, 31.8, 36.2, 113.2, 117.3, 120.5, 120.7, 125.4, 127.9, 129.7, 130.7, 133.1, 133.5, 143.4, 143.7. ^19^F{^1^H} NMR (376 MHz, CDCl_3_, rt): *δ* 182.5. Anal. Calcd for C_17_H_18_BrF_3_O_3_S: C, 46.48; H, 4.13%. Found: C, 46.72; 4.20%. 

#### 3.3.5. Synthesis of 3-Bromo-6-hexyl-2-(2-trimethylsilylethynyl)naphthalene (**6**)

To a solution of **5** (2.54 g, 5.78 mmol, 1 equiv) in DMF (16 mL) in a 50 mL of Schlenk tube, were added trimethylsilylethyne (0.85 mL, 6.01 mmol, 1.0 equiv), PdCl_2_(PPh_3_)_2_ (203 mg, 0.289 mmol, 5 mol %), CuI (110 mg, 0.58 mmol, 10 mol %), and triethylamine (16 mL). The resulting mixture was stirred at room temperature for 14 h. The reaction mixture was quenched with 1 M HCl, and extracted with CH_2_Cl_2_. The combined organic layers were washed with brine, and dried over MgSO_4_. After the volatiles were evaporated (400 Torr, 40 °C), the residue was purified by silica gel column chromatography (hexane) to give **6** (1.95 g, 5.03 mmol) in 87% yield as a yellow solid. *R*_f_ = 0.53. Mp 58–59 °C. FT-IR (KBr, cm^−1^): 2928 (s), 2853 (m), 2154 (m). ^1^H NMR (400 MHz, CDCl_3_, rt): *δ* 0.30 (s, 9H), 0.88 (t, *J* = 6.9 Hz, 3H), 1.30–1.38 (m, 6H), 1.65–1.70 (m, 2H), 2.74 (t, *J* = 7.7 Hz, 2H), 7.33 (dd, *J* = 8.4 Hz, *J* = 1.5 Hz, 1H), 7.47 (s, 1H), 7.66 (d, *J* = 8.4 Hz, 1H), 7.99 (d, *J* = 2.4 Hz, 2H); ^13^C{^1^H} NMR (150 MHz, CDCl_3_, rt): *δ* 0.05, 14.2, 22.7, 29.1, 31.3, 31.8, 36.3, 98.9, 103.7, 121.6, 122.0, 125.3, 127.6, 128.6, 130.2, 130.6, 133.5, 134.1, 142.9. Anal. Calcd for C_21_H_27_BrSi: C, 65.10; H, 7.02%. Found: C, 65.11; H, 7.03%. 

#### 3.3.6. Synthesis of 6-Hexylnaphtho[2,3-*b*]thiophene (**7**)

To a 50 mL of Schlenk tube containing **6** (410 mg, 1.06 mmol), were added sodium sulfide anhydrate (822 mg, 3.42 mmol, 3.2 equiv) and NMP (30 mL). The resulting mixture was stirred at 195 °C (salt bath) for 12 h. The reaction mixture was quenched with sat. NH_4_Cl aq. The precipitate was filtered and washed with water. The crude mixture was purified by silica gel column chromatography (hexane) to give **7** (267 mg, 0.90 mmol) in 85% yield as a white solid. *R*_f_ = 0.50. Mp 120–121°C. FT-IR (KBr, cm^−1^): 2920 (s), 2868 (m). ^1^H NMR (300 MHz, CDCl_3_, rt): *δ* 0.89 (t, *J* = 7.1 Hz, 3H), 1.30–1.38 (m, 6H), 1.70–1.75 (m, 2H), 2.79 (t, *J* = 7.8 Hz, 2H), 7.32 (dd, *J* = 8.6 Hz, *J* = 1.7 Hz, 1H), 7.43 (dd, *J* = 5.7 Hz, *J =* 18.3 Hz, 2H), 7.66 (s, 1H), 7.89 (d, *J* = 8.7 Hz, 1H), 8.28 (d, *J* = 4.8 Hz, 2H); ^13^C{^1^H} NMR (150 MHz, CDCl_3_, rt): *δ* 14.3, 22.8, 29.2, 31.3, 31.9, 36.4, 120.1, 121.7, 123.6, 125.4, 127.0, 127.6, 128.2, 129.7, 131.4, 138.4, 140.1. Anal. Calcd for C_18_H_20_S: C, 80.55; H, 7.51%. Found: C, 80.20; H, 7.49%. 

#### 3.3.7. Synthesis of 2,5-Bis(7-hexylnaphtho[2,3-*b*]thiophen-2-yl)benzendicarboxaldehyde (**9**)

To a solution of **7** (86.0 mg, 0.29 mmol, 2.2 equiv) in dehydrated THF (2.5 mL) in 20 mL of Schlenk tube, was added dropwise *n*-BuLi (1.6 M in hexane, 0.2 mL, 0.32 mmol, 2.4 equiv) at −78 °C. The resulting mixture was stirred at room temperature. After 1 h, ZnCl_2_ (1.0 M in THF solution, 0.3 mL, 0.32 mmol, 2.3 equiv) was added to at 0 °C, and the reaction mixture was stirred at room temperature for 1 h. Then, Pd(dba)_2_ (3.7 mg, 0.0065 mmol, 5 mol %), HP*^t^*Bu_3_·BF_4_ (3.0 mg, 0.013 mmol, 10 mol %), and **8** (50.0 mg, 0.13 mmol) were added and the reaction mixture was refluxed for 3 h. The reaction mixture was cooled to room temperature, quenched with water, and poured into MeOH. The precipitates were filtered and washed with hot MeOH (30 mL). The residue was purified by silica gel column chromatography (hexane:CHCl_3_ = 1:1) to give **9** (64.3 mg, 0.10 mmol) in 74% yield as an orange solid. *R*_f_ = 0.75. Mp 278-279 °C. FT-IR (KBr, cm^−1^): 2926 (s), 2852 (m), 1683 (s). ^1^H NMR (600 MHz, CDCl_3_, rt): *δ* 0.90 (t, *J* = 6.9 Hz, 6H), 1.32–1.43 (m, 12H), 1.72–1.77 (m, 4H), 2.82 (t, *J* = 7.5 Hz, 4H), 7.38 (dd, *J* = 8.4 Hz, *J* = 1.2 Hz, 2H), 7.43 (s, 2H), 7.70 (s, 2H), 7.78 (d, *J* = 9 Hz, 2H), 7.82 (d, *J* = 13.8 Hz, 4H), 8.34 (s, 2H), 10.41 (s, 2H); ^13^C{^1^H} NMR (150 MHz, CDCl_3_, rt): *δ* 14.1, 22.6, 29.0, 31.1, 31.7, 36.2, 119.8, 122.5, 125.3, 126.9, 127.4, 128.2, 129.9, 130.9, 131.8, 137.1, 137.6, 137.9, 138.3, 138.7, 140.8, 190.7. Anal. Calcd for C_44_H_42_O_2_S_2_: C, 79.24; H, 6.35%. Found: C, 78.90; H, 6.28%. 

#### 3.3.8. Synthesis of 2,2′-(2,5-Bis(7-hexylnaphtho[2,3-*b*]thiophen-2-yl)-1,4-phenylene)bis(oxirane) (**10**)

To a 20 mL of Schlenk tube containing potassium hydroxide (92.6 mg, 1.65 mmol, 5.5 equiv), was added benzonitrile (10 mL). The reaction mixture was stirred at room temperature. After 20 min, **9** (200.0 mg, 0.3 mmol) and trimethylsulfonium iodide (147.0 mg, 0.72 mmol, 2.4 equiv) were added, and the resulting mixture was stirred at 65 °C for 4 h. The reaction mixture was returned to room temperature, quenched with water, and added MeOH (5 mL). The precipitates were filtered, and washed with water, MeOH, and hexane successively to give **10** (201.9 mg, 0.29 mmol) in 97% yield as a yellow solid. Mp 237–239°C. FT-IR (KBr, cm^−1^): 2924 (s), 2852 (m), 1249 (m), 885 (s), 810 (m). ^1^H NMR (600 MHz, CDCl_3_, rt): *δ* 0.90 (t, *J* = 6.8 Hz, 6H), 1.32–1.41 (m, 12H), 1.72–1.77 (m, 4H), 2.813 (t, *J* = 7.5 Hz, 4H), 2.89–2.98 (m, 2H), 3.25–3.28 (m, 2H), 4.23–4.26 (m, 2H), 7.43 (dd, *J* = 8.7 Hz, *J* = 1.5 Hz, 2H), 7.49 (s, 2H), 7.64 (d, *J* = 1.5 Hz, 2H), 7.68 (s, 2H), 7.90 (d, *J* = 8.7 Hz, 2H), 8.27 (d, *J* = 4.5 Hz, 4H); ^13^C{^1^H} NMR (150 MHz, CDCl_3_, rt): *δ* 14.2, 22.8, 29.2, 31.3, 31.9, 36.4, 50.9, 51.8, 119.7, 122.0, 123.9, 125.5, 126.8, 127.0, 127.2, 128.2, 130.0, 131.6, 134.5, 135.8, 138.9, 140.5, 141.6. HRMS (EI^+^ and FAB^+^) was not detected. 

#### 3.3.9. Synthesis of 4,14-dihexyldinaphto[2,3-*d*:2′,3′-*d*’]anthra[1,2-*b*:5,6-*b’*]dithiophene (C6-DNADT)

To a 50 mL of Schlenk tube containing **10** (77.7 mg, 0.11 mmol), were added indium(III) chloride anhydrous (5.7 mg, 0.02 mmol, 20 mol %) and dehydrated 1,2-dichloroethane (30 mL). The reaction mixture was stirred at reflux temperature for 48 h. After the mixture was returned to room temperature, MeOH was added. The precipitates were filtered, and washed with water, MeOH, and hexane successively. The residue was purified by vacuum sublimation (source temperature, 390 °C under 10^-3^ Pa) to give C6-DNADT (13.9 mg, 0.02 mmol) in 19% yield as an orange solid. Mp >300 °C. FT-IR (KBr, cm^−1^): 2924 (s), 2852 (m). The ^1^H and ^13^C{^1^H} NMR spectra were not obtained owing to its poor solubility. Anal. Calcd for C_46_H_42_S_2_: C, 83.84; H, 6.42%. Found: C, 83.65; H, 6.23%. 

### 3.4. Fabrication of Vapor-Deposited OFET Devices 

Typical bottom-gate top-contact OFET devices were fabricated as follows: All processes were performed under a nitrogen atmosphere except for substrate cleaning. A heavily doped n-Si wafer with a 200 nm-thick thermally grown SiO_2_ (*C*_i_ = 17.3 nF cm^−2^) as the dielectric layer was used as the substrate. The n^+^-Si/SiO_2_ substrates were carefully cleaned by ultrasonication with acetone and isopropanol for 10 min, respectively. After drying, the substrates were irradiated with UV−O_3_ for 20 min and then treated with a solution of 0.1 M *n*-octyltrichlorosilane (OTS) or *n*-octadecyltrichlorosilane (ODTS) in anhydrous toluene to form the self-assembled monolayer (SAM). The active layers were deposited on the SAM treated substrate by vapor deposition at a rate of 0.5 Å s^−1^ under reduced pressure (5 × 10^−5^ Pa). The substrate temperature was room temperature. Thermal annealing was performed at 50, 100, and 150 °C for 30 min on the hotplate in the glovebox. After treatment, gold electrodes (67 nm-thick) were deposited through a shadow mask on top of the active layer under reduced pressure (5 × 10^−5^ Pa). The current−voltage characteristics of the OFETs were measured at room temperature in air on a Keithley 6430 sub-femto ampere remote source meter combined with a Keithley 2400 measure-source unit. Field effect mobilities were calculated in the saturation regime of *I*_D_ using the following equation (1), where *C*_i_ is the capacitance of the SiO_2_ insulator; *I*_D_ is the source−drain current; and *V*_D_, *V*_G_, and *V*_th_ are the source−drain, gate, and threshold voltages, respectively. The current on/off ratio (*I*_on_/*I*_off_) was determined from a minimum *I*_D_ at around *V*_G_ = 0−10 V and maximum *I*_D_ at *V*_G_ = −60 V.

*I*_D_ = (*WC*_i_/2*L*)*μ*(*V*_G_ − *V*_th_)^2^ (1)

## 4. Conclusions

In summary, we have designed the molecule by DFT calculation and found that HOMO and NHOMO levels can be controlled by the installation of alkyl chains onto the framework. Hence, the DNADT derivative, C6-DNADT, bearing two hexyl chains along the longitudinal molecular axis have successfully been synthesized. C6-DNADT has similar optical energy gap of 2.48 eV to that of DNADT and sufficiently deep HOMO energy level of −5.29 eV, which is a close value to the work function of gold, implying the high air-stability and the smooth hole injection in OFETs. Furthermore, C6-DNADT also has the high thermal stability even in the existence of flexible alkyl chains. From AFM and GIWAXS analyses, although the introduction of two hexyl groups along the molecular long-axis direction can improve the molecular orientation, the crystallinity of C6-DNADT in thin film was much poorer than that of DNADT. This may be due to the shorter length of alkyl side chains than that of the central DNADT framework, which may suppress a fastener effect. As the result, the fabricated devices based on the C6-DNADT polycrystalline film exhibited the maximum hole mobility of up to 2.6 × 10^−2^ cm^2^ V^−1^ s^−1^, which was much lower than that of our previously reported DNADT. From these results, the introduction of optimal alkyl chains is highly important to develop the high-performance materials for FETs. Currently, the synthesis and characterization of DNADT derivatives by installing longer alkyl groups are elucidated for improving OFET properties, expecting a more suitable packing structure in the solid state due to tunable intermolecular hydrophobic interactions. This study provides a potential avenue to be explored in the design of organic molecules suitable for FET materials. 

## Figures and Tables

**Table 1:** OFET Characteristics of C6-DNADT. (Thickness: 50 nm).

SAM	*T*_anneal_ / °C ^a^	*µ*_max_ / cm^2^ V^–1^ s^–1 b^	*V*_th_/V	*I*_on_/*I*_off_
OTS	as depo.	1.9 × 10^−2^	−9	10^4^–10^5^
	50	2.1 × 10^−2^	−13	10^4^–10^5^
	100	2.6 × 10^−2^	−10	10^4^–10^5^
	150	2.1 × 10^−2^	−12	10^4^–10^5^
ODTS	as depo.	1.4 × 10^−2^	−22	10^4^–10^5^
	50	7.7 × 10^−3^	−5	10^4^–10^5^
	100	6.9 × 10^−3^	−5	10^4^–10^5^
	150	4.4 × 10^−3^	−2	10^4^–10^5^

^a^ Thermal annealing process was carried out at 50, 100, and 150 °C for 30 min under an inert atmosphere; ^b^ Calculated with the saturated regime.

## Supplementary Materials

The following are available online at https://www.mdpi.com/1422-0067/21/7/2447/s1. ^1^H and ^13^C{^1^H} NMR spectra of the final products.

## Abbreviations

OFET	Organic field-effect transistor
C6-DNADT	4,14-Dihexyldinaphtho[2,3-*d*:2’,3’-*d*’]anthra[1,2-*b*:5,6-*b*’]dithiophene
GIWAXS	Grazing incidence wide-angle X-ray scattering
AFM	Atomic force microscopy
HOMO	Highest occupied molecular orbital
C8-BTBT	2,7-Dioctyl[1]benzothieno[3,2-*b*][1]benzothiophene
XRD	X-ray diffraction
DFT	Density functional theory
NHOMO	Second (next) highest occupied molecular orbital
DMF	*N*,*N*-Dimethylformamide
dppf	1,1’-Bis(diphenylphosphino)ferrocene
THF	Tetrahydrofuran
TMS	Trimethylsilyl
DCE	1,2-Dichloroethane
NMP	*N*-Methyl-2-pyrrolidone
NMR	Nuclear magnetic resonance
UV	Ultraviolet
TGA	Thermogravimetric analysis
DSC	Differential scanning calorimetry
OTS	*n*-Octyltrichlorosilane
ODTS	*n*-Octadecyltrichlorosilane
SAM	Self-assembled monolayer
RMS	Root-mean-square
TLC	Thin layer chromatography
HRMS	High-resolution mass spectrometry
FT-IR	Fourier transform infrared spectroscopy
TCI	Tokyo Chemical Industry Co., Ltd.
FAB	Fast atom bombardment
EI	Electron impact
equiv	Equivalent
sat.	Saturated
aq.	Aqueous
