# Supplementary material for: Synthesis of Dinaphtho[2,3-d:2’,3’-d’]anthra[1,2-b:5,6-b’]dithiophene (DNADT) Derivatives: Effect of Alkyl Chains on Transistor Properties
Source: Int J Mol Sci. 2020 Apr 1;21(7):2447. doi: 10.3390/ijms21072447 (PMC7177802; doi:10.3390/ijms21072447)
Supplement: Supplementary file 1 [file ijms-21-02447-s001.pdf]

Supplementary Materials

Synthesis of Dinaphtho[2,3-*d*:2',3'-*d*']anthra[1,2-*b*:5,6-*b*']dithiophene (DNADT) Derivatives: Effect of Alkyl Chains on Transistor Properties

Takumi Ishida [1], Yuta Sawanaka [1], Ryota Toyama [1], Zhenfei Ji [1], Hiroki Mori [2] and Yasushi Nishihara [2,*]

[1] Graduate School of Natural Science and Technology, Okayama University, 3-1-1 Tsushimanaka, Kita-ku, Okayama 700-8530, Japan; pimf2p6f@s.okayama-u.ac.jp (T.I.); pyhb7jum@s.okayama-u.ac.jp (Y.S.); pnah56kw@s.okayama-u.ac.jp (R.T.); puf77a9l@s.okayama-u.ac.jp (Z.J.)

[2] Research Institute for Interdisciplinary Science, Okayama University, 3-1-1 Tsushimanaka, Kita-ku, Okayama 700-8530, Japan; h-mor@okayama-u.ac.jp (H.M.)

* Correspondence: ynishiha@okayama-u.ac.jp; Tel.: +81-86-251-7855

Copies of NMR Spectra

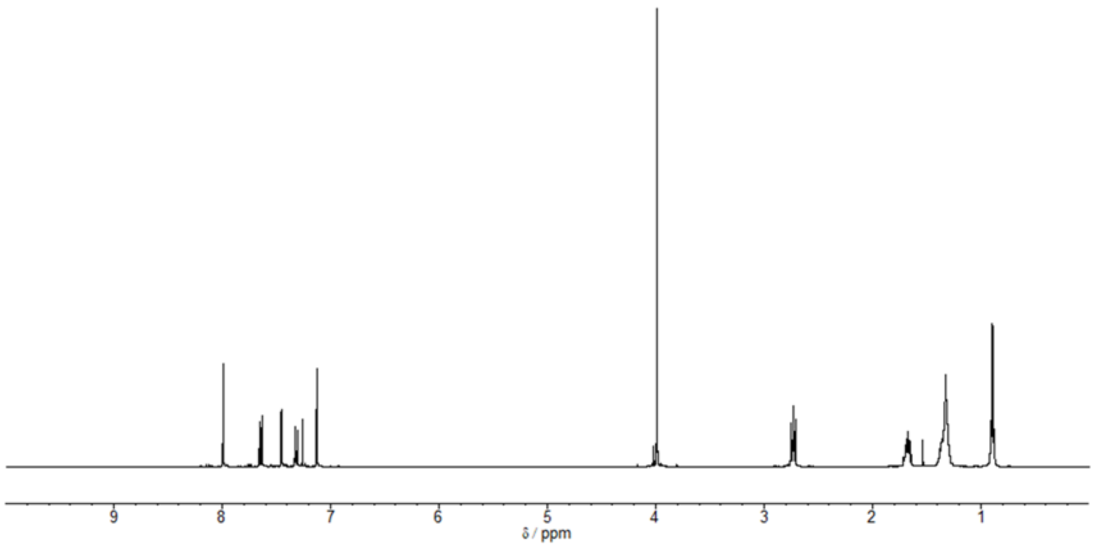

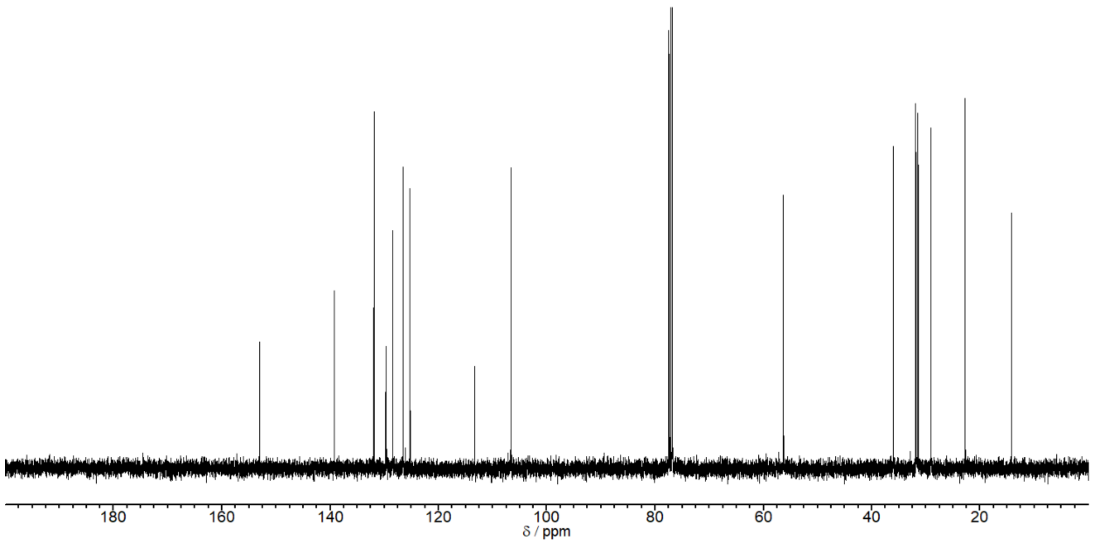

^{1}H NMR (400 MHz), and $^{13}C\{^{1}H\}$ NMR (150 MHz) spectra of **3** ($CDCl_3$, rt).

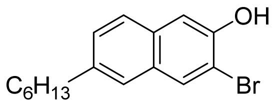

4

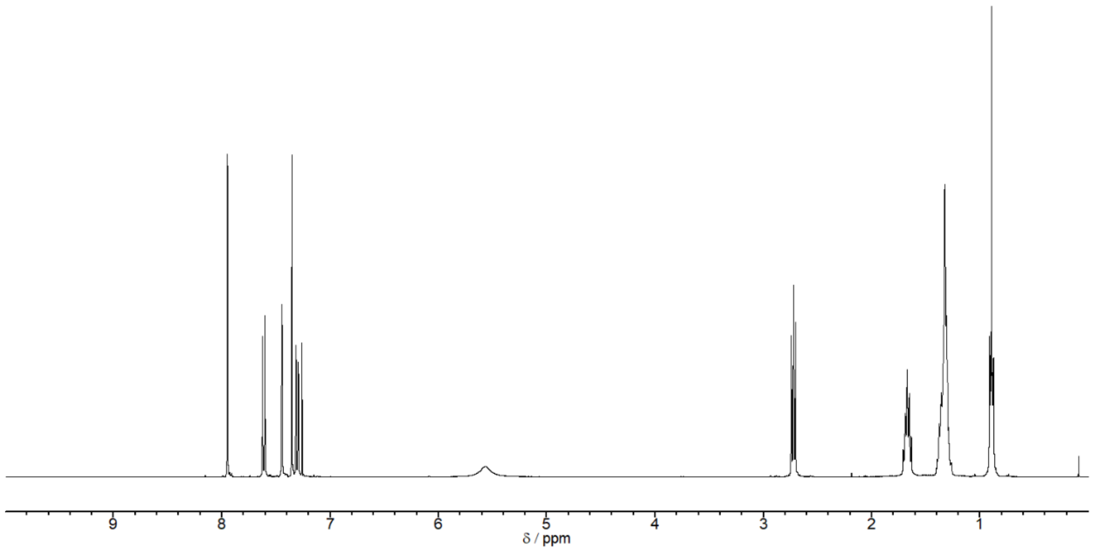

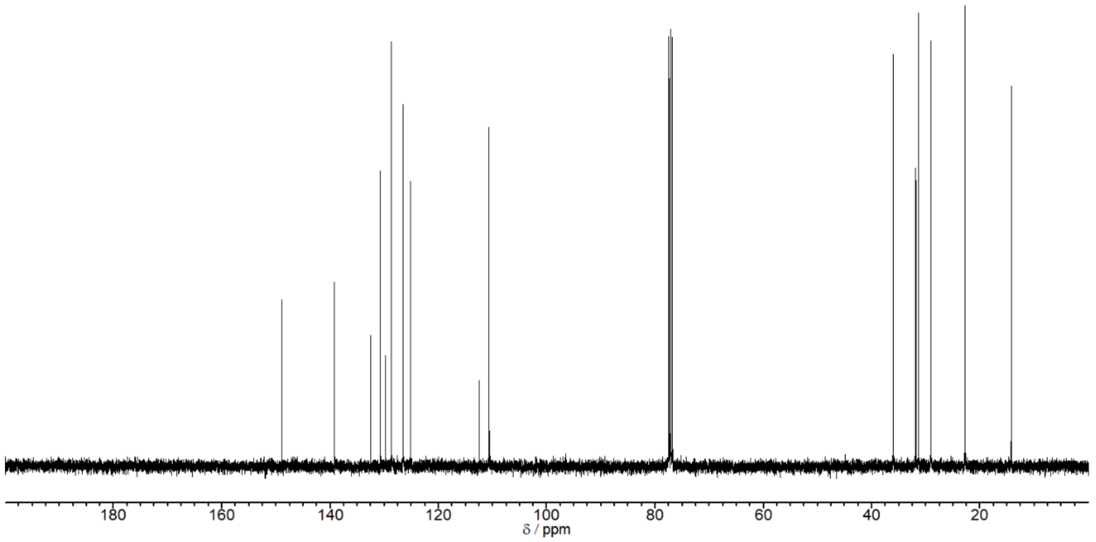

^{1}H NMR (400 MHz), and $^{13}C\{^{1}H\}$ NMR (150 MHz) spectra of **4** ($CDCl_3$, rt).

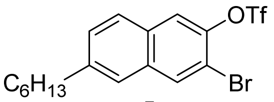
OTf
Br
C_6H_{13}

5

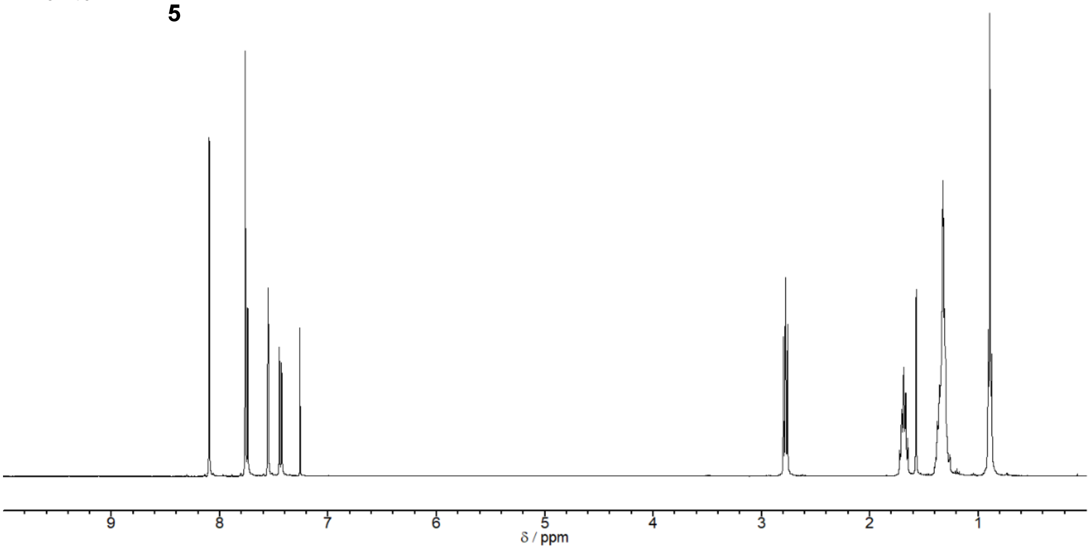
9
8
7
6
5
4
3
2
1
δ / ppm

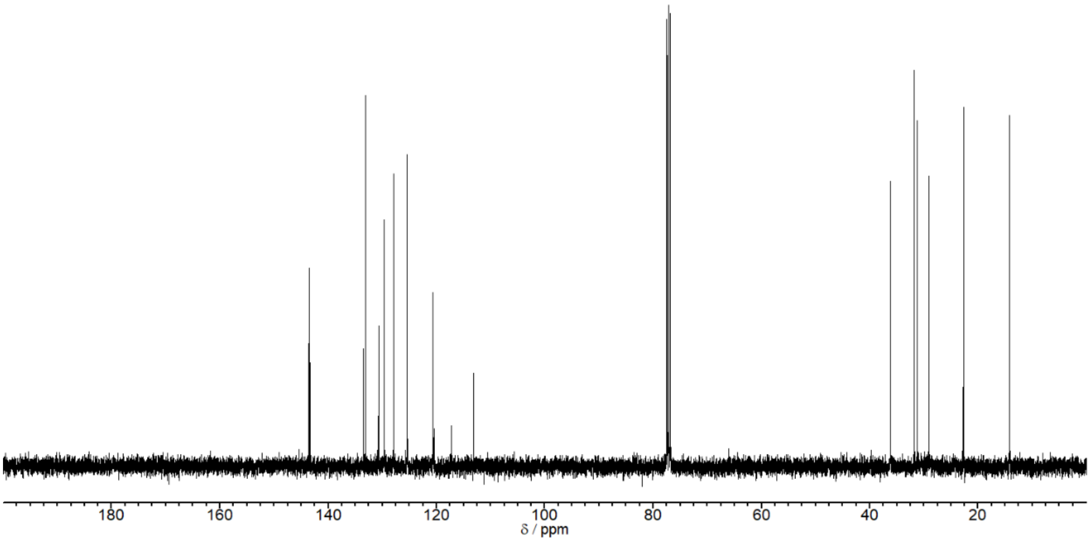
180
160
140
120
100
80
60
40
20
δ / ppm

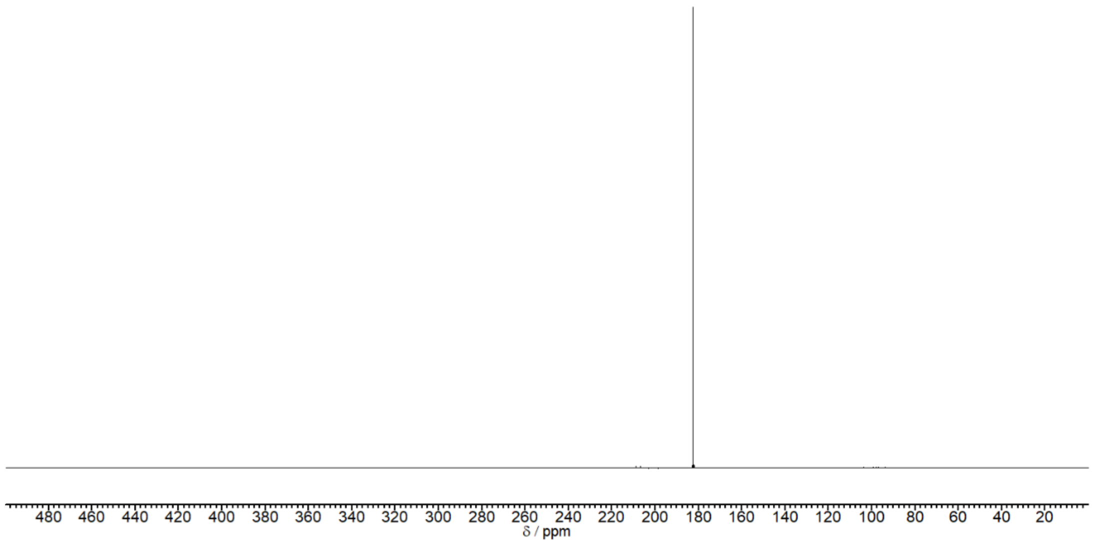

^{1}H NMR (600 MHz), ^{13}C{^{1}H} NMR (150 MHz) and ^{19}F{^{1}H} NMR (376 MHz) spectra of **5** ($CDCl_3$, rt).

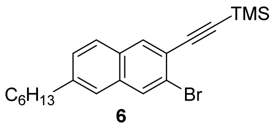

6

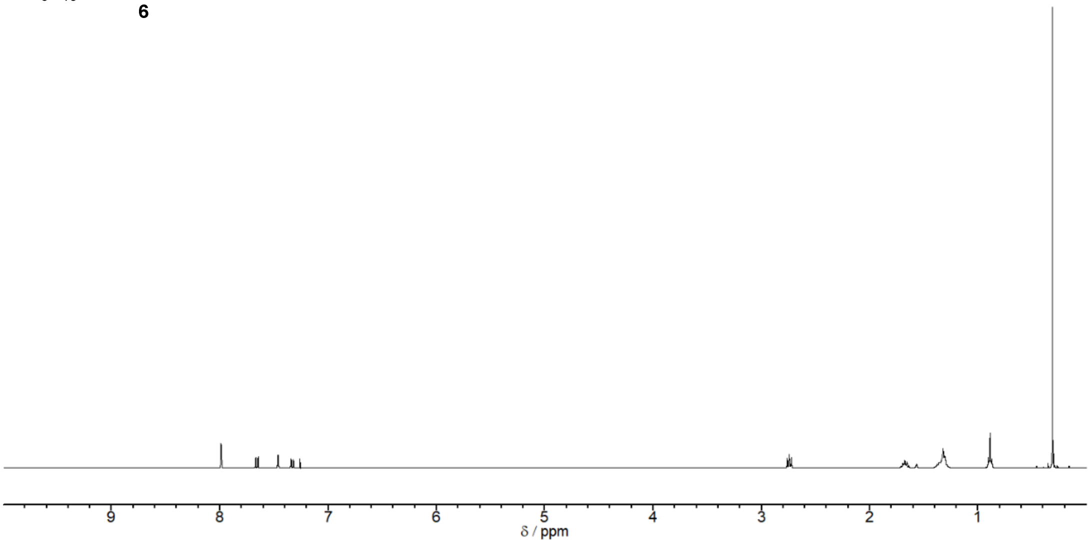

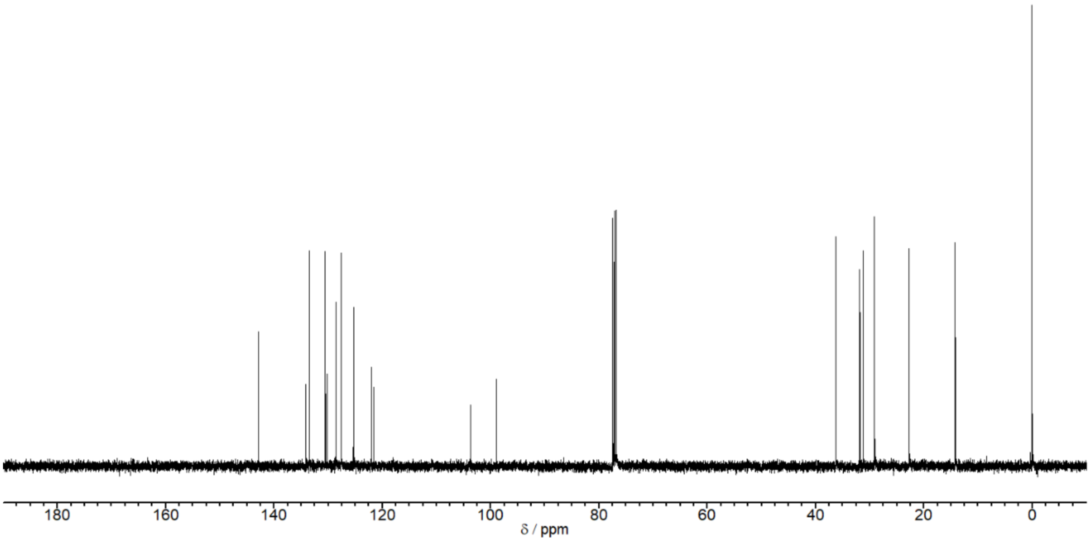

^{1}H NMR (400 MHz), and ^{13}C{^{1}H} NMR (150 MHz) spectra of **6** ($CDCl_3$, rt).

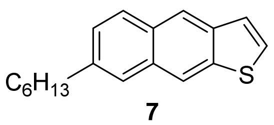

7

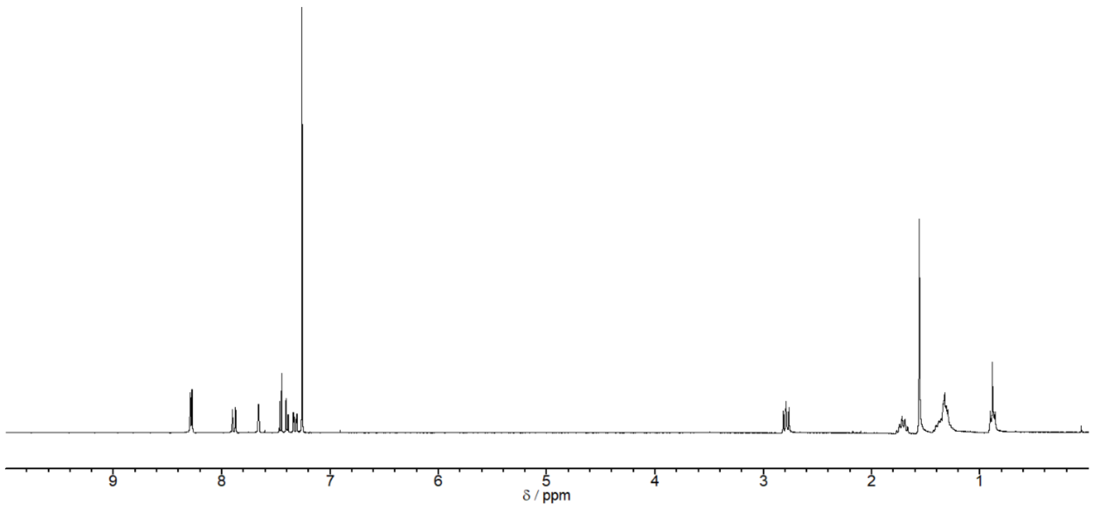

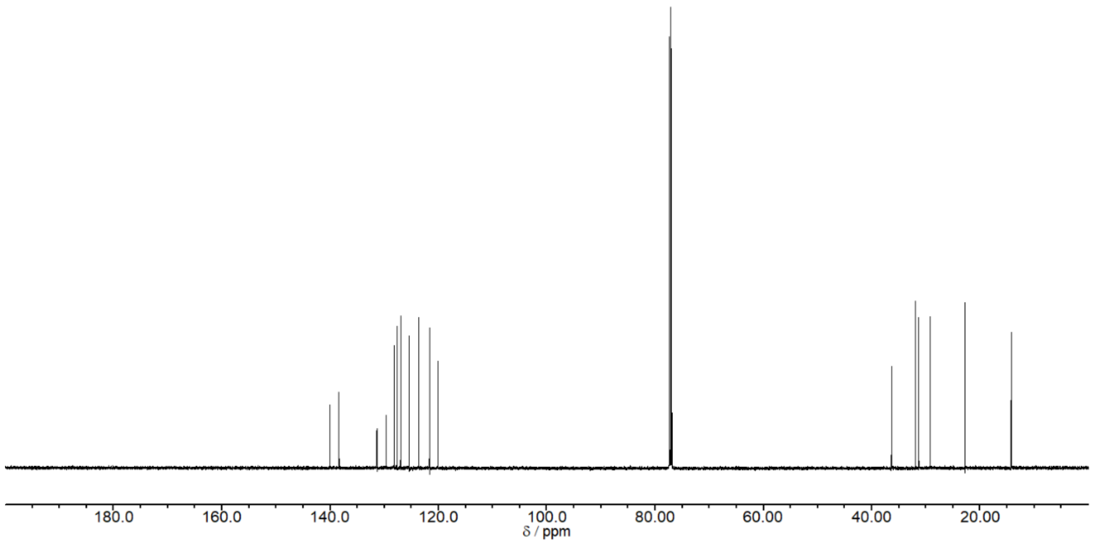

^{1}H NMR (300 MHz) and $^{13}C\{^{1}H\}$ NMR (150 MHz) spectra of **7** ($CDCl_3$, rt).

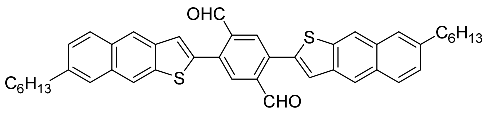

9

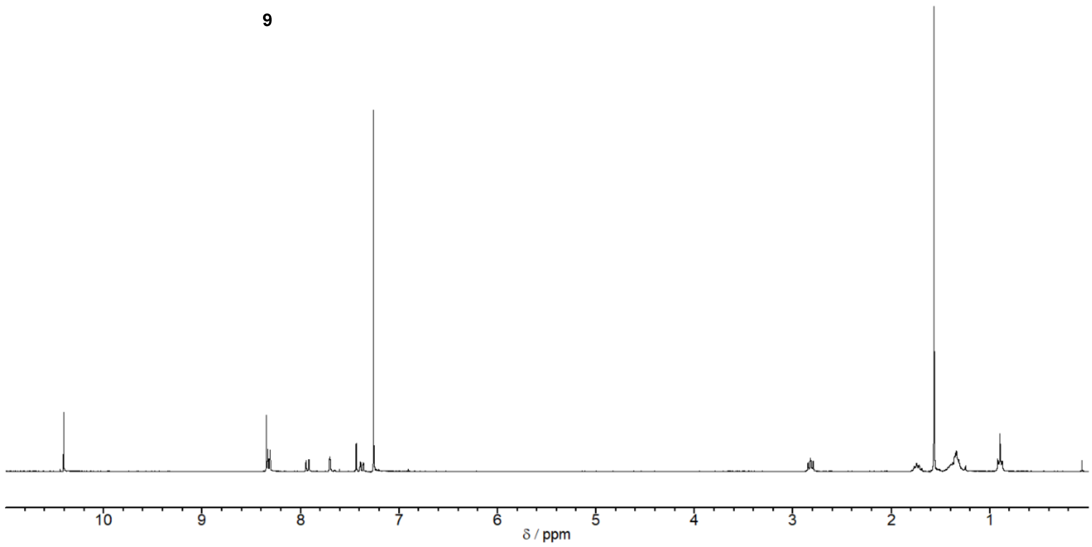

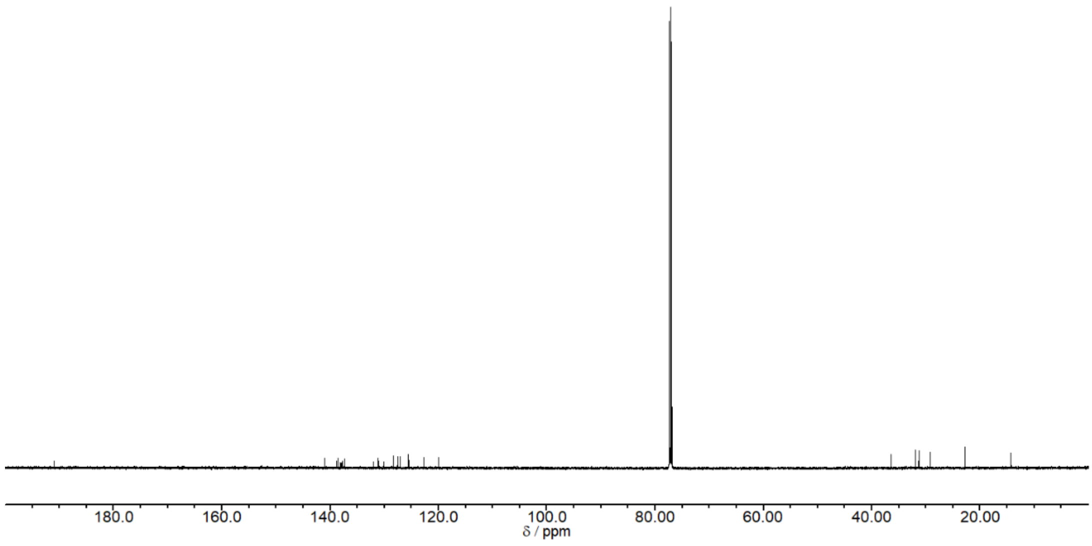

^{1}H NMR (600 MHz), and $^{13}C\{^{1}H\}$ NMR (150 MHz) spectra of **9** ($CDCl_3$, rt).

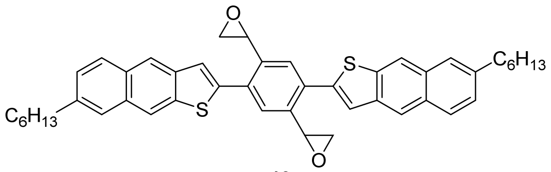

10

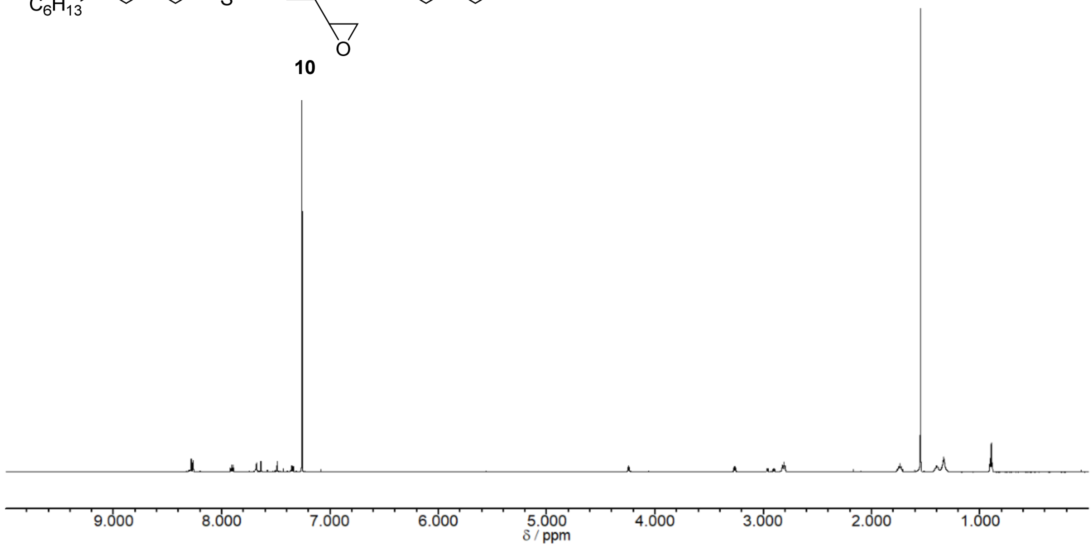

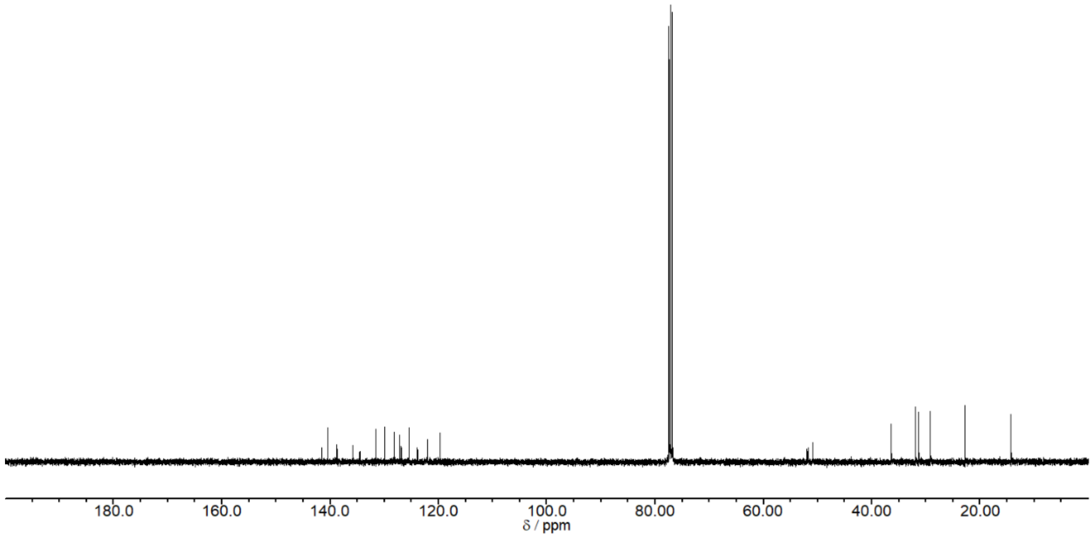

^{1}H NMR (600 MHz), and ^{13}C{^{1}H} NMR (150 MHz) spectra of **10** ($CDCl_3$, rt).
